# Association between metabolic syndrome components and the risk of malignant neoplasms of the brain: a nationwide cohort study

**DOI:** 10.3389/fonc.2026.1868270

**Published:** 2026-06-18

**Authors:** Taek Min Nam, Min-ho Kim, Na Rae Yang, Yongjae Cho, Sung-Kyun Hwang, Dosang Cho, Young Goo Kim

**Affiliations:** 1 Department of Neurosurgery, Mokdong Hospital, College of Medicine, Ewha Womans University, Seoul, Republic of Korea; 2 Informatization Department, Seoul Hospital, College of Medicine, Ewha Womans University, Seoul, Republic of Korea; 3 Department of Neurosurgery, Seoul Hospital, College of Medicine, Ewha Womans University, Seoul, Republic of Korea

**Keywords:** epidemiology, malignant neoplasms of the brain, metabolic syndrome, primary brain tumors, triglycerides

## Abstract

**Objective:**

To examine the relationship between metabolic syndrome (MetS) components and the development of malignant neoplasms of the brain (MNBs) and to identify the most significant influence.

**Methods:**

We conducted a nationwide cohort study using the Korean National Health Insurance Service database, enrolling 3, 976, 961 individuals aged ≥40 years who completed a national health checkup in 2009. Participants were followed from 2010 to 2020 to assess MNB incidence using International Classification of Diseases, 10th Revision, codes C71.0–C71.9. Participants were stratified by the number of MetS components (0–5). Cox proportional hazards models were used to estimate hazard ratios (HRs), adjusted for age, sex, smoking, alcohol use, exercise, and comorbidities.

**Results:**

In multivariate analysis adjusting for age, sex, lifestyle factors, and comorbidities, individual component counts modeled as discrete categorical comparisons against the zero-component reference did not reach statistical significance at any level (HR for 5 components: 1.070; 95% CI: 0.941–1.217; p = 0.303). However, when participants were stratified by clinically established cumulative thresholds, those with ≥3 MetS components demonstrated a modestly but statistically significantly increased MNB risk (HR: 1.091; 95% CI: 1.017–1.170; ≥5 components, 1.153; 95% CI: 1.040–1.278). Among individual components, elevated triglycerides showed the strongest independent association with MNB risk (adjusted HR: 1.517; 95% CI: 1.179–1.953), whereas the other components showed weaker or non-significant associations.

**Conclusions:**

The cumulative burden of MetS was modestly but statistically significantly associated with MNB risk, with elevated triglycerides showing the strongest association. These findings suggest that metabolic health management, particularly lipid monitoring, may inform brain tumor risk assessment. Critically, however, the outcome variable encompasses a diagnostically heterogeneous group of brain malignancies identified by administrative ICD-10 coding, and no mechanistic inference or histological subtype-specific conclusion can be drawn from the present data. These findings should be considered hypothesis-generating and require prospective validation using pathologically and molecularly characterized tumor registries before any clinical recommendations regarding lipid management for brain tumor prevention can be made.

## Introduction

Metabolic syndrome (MetS) is a cluster of interrelated conditions, including central obesity, hypertension, dyslipidemia, and insulin resistance, that collectively increase the risk of cardiovascular disease and type 2 diabetes (1, 2). The diagnosis of MetS is confirmed when three or more of the following criteria are met: i) waist circumference >102 cm in men or >88 cm in women; ii) serum triglyceride level ≥150 mg/dL; iii) reduced high-density lipoprotein (HDL) cholesterol level, defined as <40 mg/dL in men or <50 mg/dL in women; iv) fasting glucose level ≥100 mg/dL; and v) systolic blood pressure ≥130 mm Hg or diastolic blood pressure ≥85 mm Hg (1, 3). The global prevalence of MetS has risen dramatically in recent decades, paralleling increases in obesity and sedentary lifestyles, and currently affects approximately 20-25% of the adult population worldwide (4).

Beyond its well-established cardiovascular and metabolic consequences, accumulating evidence has linked MetS to an increased risk of various malignancies, including prostate cancer, colorectal cancer, and breast cancer recurrence. Meta-analyses have shown that individuals with MetS have a 1.3- to 2.0-fold increased risk of developing these cancers compared with those without MetS (5–8). However, the relationship between MetS and malignant neoplasms of the brain (MNBs) remains inadequately characterized.

MNBs, particularly glioblastoma (GBM), are among the most rapidly progressive and fatal intracranial malignant tumors (9). These tumors are characterized by rapid growth, invasiveness, and resistance to conventional therapies. Although progress has been made in understanding the pathophysiology of brain malignancies, clinical outcomes remain poor across most histological subtypes (10). For example, patients with GBM who undergo standard treatment with surgery followed by concurrent chemoradiotherapy and adjuvant temozolomide typically have a median overall survival of approximately 14.6 months, and few survive more than 2 years (11, 12).

Emerging research has examined the relationship between MetS and brain malignancies. Some retrospective studies have reported a higher prevalence of MetS among patients with GBM than in the general population, although this association may be influenced by age and other confounding factors (13). Accumulation of metabolic risk factors has been linked to survival outcomes in some cohorts, with hyperglycemia identified as a potential risk factor in selected analyses (13). Some evidence suggests that metabolic dysfunction may be associated with outcomes in patients with brain malignancies receiving standard treatment (14). However, most prior research has focused on the prevalence of MetS among patients already diagnosed with brain tumors or its impact on survival, rather than examining MetS as a potential risk factor for tumor development. Thus, this study aimed to investigate the relationship between MetS components and the development of MNBs as defined by ICD-10 coding, and to identify which metabolic factors show the strongest associations.

## Methods

This retrospective study used data from the South Korean National Health Insurance Service (NHIS) insurance claims database from January 2010 to December 2020. The study was approved by the Institutional Review Board (IRB) of Ewha Womans University (IRB No. 2025-06-004) and conducted in accordance with the Declaration of Helsinki. Given the retrospective design and the use of anonymized datasets, informed consent was waived.

The NHIS, a mandatory health insurance program, covers nearly the entire Korean population—approximately 97%—with the remainder supported by the Medical Aid Program. The Health Insurance Review and Assessment Service (HIRA) manages nationwide medical claims and provides extensive datasets that include demographic characteristics, clinical diagnoses, prescriptions, medical procedures, and health service utilization. Diagnoses are coded using the Korean Standard Classification of Diseases, 7th Revision (KCD-7), a modified version of the ICD-10. All Korean citizens are assigned a unique resident registration number, enabling consistent and accurate linkage of medical records across healthcare facilities. This system ensures data integrity and prevents duplication, even when individuals seek care at multiple institutions. Furthermore, death registration is legally mandatory and includes documentation of the cause of death before funeral proceedings, enabling reliable mortality tracking within the national databases. The national health checkup program in South Korea is an essential service designed for the early detection of diseases, such as cancer, and for assessing lifestyle-related risk factors, promoting better health management. The program provides general checkups to the public every 2 years.

For this study, participants aged ≥40 years with health checkup data recorded in the NHIS database in 2009 were included. We assessed whether patients developed MNBs from 2010 to 2020. A total of 4, 361, 892 individuals received routine health checkups in 2009. Of these, 188, 551 were excluded owing to missing data. The NHIS data used in this study were applied to HIRA for research purposes, and there was no selection bias because individuals with missing values were excluded at the application stage. Additionally, 195, 515 individuals were excluded because they had a prior MNB diagnosis. Furthermore, 865 individuals who died or were newly diagnosed with MNB within one year of baseline were also excluded. A total of 3, 976, 961 participants were finally enrolled in this study. Each participant was categorized from 0 to 5 based on the number of MetS components. The primary outcome was the development of MNBs during the follow-up period, identified using ICD-10 codes C71.0–C71.9 (**Table 1**).

**Table 1:** ICD-10 codes for malignant neoplasms of the brain.

Codes	Malignant neoplasms of the brain
C71.0	Cerebrum, except lobes and ventricles; supratentorial NOS
C71.1	Frontal lobe
C71.2	Temporal lobe
C71.3	Parietal lobe
C71.4	Occipital lobe
C71.5	Cerebral ventricle, exclude forth ventricle
C71.6	Cerebellum
C71.7	Brain stem; fourth ventricle, infratentorial NOS
C71.8	Overlapping lesion of the brain
C71.9	Brain, unspecified

ICD-10, International Classification of Diseases; Tenth Revision; NOS.

Statistical analyses were conducted using SAS software (version 9.4, SAS Institute, Cary, NC, USA). Cox proportional hazards regression models were used to estimate hazard ratios (HRs) and 95% confidence intervals (CIs) for the association between MetS components and the risk of MNB. Both univariate and multivariate models were developed. The multivariate analysis controlled for age, sex, smoking status, alcohol consumption, exercise frequency, and comorbid conditions, including hypertension, diabetes, and hyperlipidemia. Both the categorical component-count models (**Table 2**) and the cumulative threshold models (**Table 3**) applied identical covariate sets in their multivariate specifications, adjusting for: age, sex, smoking status (never/quit/current), alcohol consumption (≤1 vs. >1 day/week), physical exercise frequency (≤3 vs. >3 days/week), hypertension, diabetes mellitus, and dyslipidemia. The same covariate structure was also applied to the individual component mandatory-inclusion analyses (**Table 4**). Given the exploratory nature of the multiple component analyses, findings should be interpreted with consideration of potential alpha inflation from multiple testing. Statistical significance was assessed using a two-sided p-value of <0.05.

**Table 2:** Hazard ratios for malignant neoplasms of the brain by the number of metabolic syndrome components.

Number of components	Univariate HR (95% CI)	p-value	Multivariate HR (95% CI)	p-value
0 (Reference)	1.00 (Reference)		1.00 (Reference)	
1	1.031 (0.946–1.124)	0.482	0.966 (0.886–1.054)	0.434
2	1.091 (1.001–1.188)	0.046	0.992 (0.908–1.085)	0.867
3	1.150 (1.052–1.257)	0.002	1.027 (0.932–1.131)	0.598
4	1.191 (1.080–1.313)	<0.001	1.044 (0.939–1.160)	0.437
5	1.313 (1.165–1.480)	<0.001	1.070 (0.941–1.217)	0.303

HR, Hazard ratio; CI, Confidence interval; Multivariate model adjusted for: age, sex, smoking status (never/quit/current), alcohol consumption (≤1 vs. >1 day/week), physical exercise (≤3 vs. >3 days/week), hypertension, diabetes mellitus, and dyslipidemia.

**Table 3:** Cumulative risk of malignant neoplasms of the brain based on metabolic syndrome component thresholds.

Threshold (≥ Components)	Univariate HR (95% CI)	p-value	Multivariate HR (95% CI)	p-value
MetS components <3 (reference)	1.00 (Reference)		1.00 (Reference)	
3	1.228 (1.167–1.291)	<0.001	1.091 (1.017–1.170)	0.015
MetS components <4 (reference)	1.00 (Reference)		1.00 (Reference)	
4	1.277 (1.205–1.354)	<0.001	1.131 (1.045–1.225)	0.002
MetS components <5 (reference)	1.00 (Reference)		1.00 (Reference)	
5	1.355 (1.235–1.486)	<0.001	1.153 (1.040–1.278)	0.007

HR, Hazard ratio; CI, Confidence interval; MetS, Metabolic syndrome; Multivariate model adjusted for: age, sex, smoking status (never/quit/current), alcohol consumption (≤1 vs. >1 day/week), physical exercise (≤3 vs. >3 days/week), hypertension, diabetes mellitus, and dyslipidemia.

**Table 4:** Effect of excluding individual metabolic syndrome components on the risk of developing malignant neoplasms of the brain.

Variable		Univariate HR (95% CI)	p-value	Multivariate HR (95% CI)	p-value
Non-metabolic syndrome (Reference)		1.00 (Reference)		1.00 (Reference)	
Metabolic syndrome	Mandatory inclusion factor				
	Waist circumference	1.204 (1.011–1.433)	0.037	1.195 (0.998–1.432)	0.053
	HDL cholesterol level	1.137 (0.932–1.387)	0.206	1.015 (0.829–1.242)	0.888
	Triglyceride level	1.740 (1.357–2.230)	<0.001	1.517 (1.179–1.953)	0.001
	Hypertension	1.170 (1.071–1.279)	<0.001	1.016 (0.909–1.135)	0.781
	Fasting blood glucose level	1.171 (1.011–1.336)	0.036	1.050 (0.899–1.227)	0.537

HR, Hazard ratio; CI, Confidence interval; HDL, High-density lipoprotein.

## Results

As shown in **Table 5**, age increased progressively with the number of MetS components, rising from 57.51 years in the 0-component group to 63.35 years in the 5-component group. Body mass index (BMI) and waist circumference also increased, with BMI rising from 22.43 to 27.43 kg/m2 and waist circumference from 76.68 to 92.86 cm. Systolic and diastolic blood pressure similarly increased with the number of components, reaching 133.29 mmHg and 80.75 mmHg, respectively, in the 5-component group. Metabolic laboratory parameters followed a similar trend: fasting blood glucose levels increased from 88.13 to 121.51 mg/dL, and triglyceride levels from 88.18 to 186.76 mg/dL. HDL cholesterol showed a decreasing trend, falling from 62.63 mg/dL in the 0-component group to 50.31 mg/dL in the 5-component group. Low-density lipoprotein cholesterol levels peaked at 2–3 components before declining slightly thereafter. Regarding comorbidities, the prevalence of hypertension increased from 17.16% to 94.91%, diabetes from 5.86% to 65.17%, and dyslipidemia from 14.24% to 83.41% across the component spectrum. The incidence of malignant brain tumors showed a slight but consistent increase with the number of MetS components, rising from 0.14% in the 0-component group to 0.20% in the 5-component group. Lifestyle factors, such as smoking and alcohol consumption, showed slight variations. The percentage of never-smokers increased from 67.85% to 69.10%, whereas that of current smokers decreased. Most participants reported exercising ≤3 days per week across all groups. Additionally, the average follow-up duration decreased slightly as metabolic burden increased, from 12.05 to 11.59 years. Overall, the number of MNB cases increased from 0.14% in the 0-component group to 0.20% in the 5-component group. The average follow-up duration ranged from 12.05 ± 1.63 years (0-component group) to 11.59 ± 2.34 years (5-component group).

**Table 5:** Baseline characteristics stratified by the number of metabolic syndrome components.

Variable	Total	Number of MetS components						p-value
		0	1	2	3	4	5	
Total number	3, 976, 961	668, 444 (16.81)	875, 576 (22.02)	849, 836 (21.37)	750, 771 (18.88)	584, 572 (14.70)	247, 762 (6.23)	
Age	60.09 ± 7.68	57.51 ± 6.94	59.00 ± 7.45	59.99 ± 7.63	60.98 ± 7.68	62.28 ± 7.67	63.35 ± 7.60	<.001
Sex								
Male	1, 926, 337 (48.44)	311, 115 (46.54)	447, 520 (51.11)	424, 651 (49.97)	356, 782 (47.52)	276, 583 (47.31)	109, 686 (44.27)	<.001
Female	2, 050, 624 (51.56)	357, 329 (53.46)	428, 056 (48.89)	425, 185 (50.03)	393, 989 (52.48)	307, 989 (52.69)	138, 076 (55.73)	
Body mass index (kg/m2)	24.14 ± 3.56	22.43 ± 2.36	23.30 ± 2.61	24.10 ± 3.84	24.71 ± 4.44	25.28 ± 2.96	27.43 ± 2.77	<.001
Waist circumference (cm)	82.27 ± 8.66	76.68 ± 6.37	79.77 ± 7.47	82.23 ± 8.01	83.98 ± 8.46	85.78 ± 8.30	92.86 ± 6.40	<.001
Systolic blood pressure (mm Hg)	126.50 ± 15.80	118.19 ± 11.92	124.03 ± 14.61	127.44 ± 15.97	129.48 ± 16.13	131.62 ± 16.03	133.29 ± 15.88	<.001
Diastolic blood pressure (mm Hg)	77.99 ± 10.16	73.69 ± 8.39	76.95 ± 9.66	78.68 ± 10.28	79.60 ± 10.43	80.24 ± 10.40	80.75 ± 10.38	<.001
Fasting blood glucose (mg/dL)	102.11 ± 27.47	88.13 ± 7.45	96.61 ± 20.10	101.50 ± 25.75	106.17 ± 30.22	113.77 ± 34.28	121.51 ± 36.54	<.001
Total cholesterol (mg/dL)	201.80 ± 43.55	199.44 ± 34.82	201.78 ± 41.41	205.51 ± 43.38	204.41 ± 46.35	198.50 ± 48.66	195.34 ± 49.09	<.001
Triglyceride (mg/dL)	142.34 ± 92.67	88.18 ± 29.17	115.02 ± 63.64	149.42 ± 90.10	171.44 ± 104.68	178.72 ± 111.08	186.76 ± 112.72	<.001
High-density lipoprotein cholesterol (mg/dL)	55.86 ± 35.05	62.63 ± 30.53	58.59 ± 38.38	54.99 ± 36.15	52.73 ± 35.68	51.69 ± 33.14	50.31 ± 28.62	<.001
Low-density lipoprotein cholesterol (mg/dL)	121.23 ± 79.03	122.41 ± 72.80	124.42 ± 75.32	124.74 ± 75.92	121.15 ± 80.46	114.60 ± 89.78	110.65 ± 84.83	<.001
Smoking status								
Never	2, 636, 771 (66.30)	453, 563 (67.85)	565, 568 (64.59)	553, 088 (65.08)	500, 581 (66.68)	392, 757 (67.19)	171, 214 (69.10)	<.001
Quit	626, 285 (15.75)	90, 655 (13.56)	136, 318 (15.57)	135, 265 (15.92)	120, 477 (16.05)	100, 789 (17.24)	42, 781 (17.27)	
Current	713, 905 (17.95)	124, 226 (18.58)	173, 690 (19.84)	161, 483 (19.00)	129, 713 (17.28)	91, 026 (15.57)	33, 767 (13.63)	
Alcohol consumption								
≤1 day/week	3, 064, 985 (77.07)	529, 069 (79.15)	663, 364 (75.76)	641, 377 (75.47)	578, 552 (77.06)	456, 268 (78.05)	196, 355 (79.25)	<.001
>1 days/week	911, 976 (22.93)	139, 375 (20.85)	212, 212 (24.24)	208, 459 (24.53)	172, 219 (22.94)	128, 304 (21.95)	51, 407 (20.75)	
Physical training								
≤3 days/week	2, 928, 480 (73.64)	488, 323 (73.05)	643, 520 (73.50)	629, 479 (74.07)	554, 056 (73.80)	427, 543 (73.14)	185, 559 (74.89)	<.001
>3 days/week	1, 048, 481 (26.36)	180, 121 (26.95)	232, 056 (26.50)	220, 357 (25.93)	196, 715 (26.20)	157, 029 (26.86)	62, 203 (25.11)	
Comorbidity								
Hypertension	1, 499, 702 (37.71)	0 (0.00)	150, 237 (17.16)	264, 344 (31.11)	381, 443 (50.81)	468, 534 (80.15)	235, 144 (94.91)	<.001
Diabetes mellitus	757, 342 (19.04)	0 (0.00)	51, 286 (5.86)	98, 763 (11.62)	173, 249 (23.08)	272, 584 (46.63)	161, 460 (65.17)	<.001
Dyslipidemia	1, 039, 705 (26.14)	0 (0.00)	0 (0.00)	120, 979 (14.24)	309, 780 (41.26)	402, 297 (68.82)	206, 649 (83.41)	<.001
Development of malignant neoplasm of the brain								
No	3, 970, 888 (99.85)	667, 530 (99.86)	874, 355 (99.86)	848, 591 (99.85)	749, 586 (99.84)	583, 554 (99.83)	247, 272 (99.80)	<.001
Yes	6, 073 (0.15)	914 (0.14)	1, 221 (0.14)	1, 245 (0.15)	1, 185 (0.16)	1, 018 (0.17)	490 (0.20)	
Follow-up (years)	11.85 ± 2.01	12.05 ± 1.63	11.93 ± 1.89	11.85 ± 2.03	11.81 ± 2.09	11.67 ± 2.27	11.59 ± 2.34	<.001

MetS, Metabolic syndrome.

HRs for MNBs were evaluated by the number of MetS components present. In the univariate analysis, risk increased progressively with each additional component, starting with two components (HR = 1.091; 95% CI: 1.001–1.188; p = 0.046), which reached statistical significance. Risk continued to rise with three components (HR = 1.150; p = 0.002), four components (HR = 1.191; p <0.001), and peaked in individuals with all five components (HR = 1.313; 95% CI: 1.165–1.480; p <0.001). However, after adjusting for confounding variables, such as age, sex, lifestyle factors, and comorbidities, none of the component counts remained statistically significant in the multivariate analysis. For example, the adjusted HR for the five-component group was 1.070 (95% CI: 0.941–1.217; p = 0.303) (**Table 2**). These findings suggest that, although the unadjusted risk appears to increase with the accumulation of MetS components, this association is attenuated when other clinical and behavioral variables are considered. Therefore, while the number of MetS components may correlate with MNB risk in raw data, its independent effect requires further investigation in controlled analyses.

The absence of statistical significance in the **Table 2** multivariate models warrants explicit explanation, as it contrasts with the significant findings in **Table 3**. Both models applied identical covariate adjustments; the divergence reflects a difference in modeling strategy rather than analytical inconsistency. In **Table 2**, participants are distributed across six discrete strata, each compared individually against the zero-component reference group, substantially reducing the event count available for each pairwise comparison — for example, the five-component stratum contributed only 490 MNB events — thereby limiting statistical power after adjustment for eight covariates. In **Table 3**, participants meeting each cumulative threshold are consolidated into a single high-burden group (e.g., ≥3 components: n=1, 583, 105; 2, 938 MNB events), substantially increasing statistical power and enabling detection of the modest but consistent excess risk. Accordingly, **Tables 2**, **3** address complementary questions: **Table 2** assesses whether each incremental increase in component count independently predicts MNB risk, whereas **Table 3** assesses whether meeting the established clinical threshold for metabolic syndrome confers independent oncological risk. A clear cumulative relationship was observed between the number of MetS components and the risk of developing MNBs. When individuals had three or more metabolic abnormalities, the multivariate HR for MNB incidence was 1.091 (95% CI, 1.017–1.170; p = 0.015), indicating a statistically significant increase in risk. This risk increased further among those with four or more components, with a multivariate HR of 1.131 (95% CI: 1.045–1.225, p = 0.002). Among participants with all five components, the risk was highest, with a multivariate HR of 1.153 (95% CI: 1.040–1.278, p = 0.007) (**Table 3**). These findings highlight a graded association, with each additional metabolic component contributing to a stepwise increase in MNB risk. Importantly, the associations remained statistically significant after adjustment for potential confounding variables, suggesting that the cumulative burden of MetS may independently elevate the likelihood of brain malignancy. This pattern supports the role of metabolic dysregulation as a systemic risk factor in the development of central nervous system tumors.

**Figure 1:**
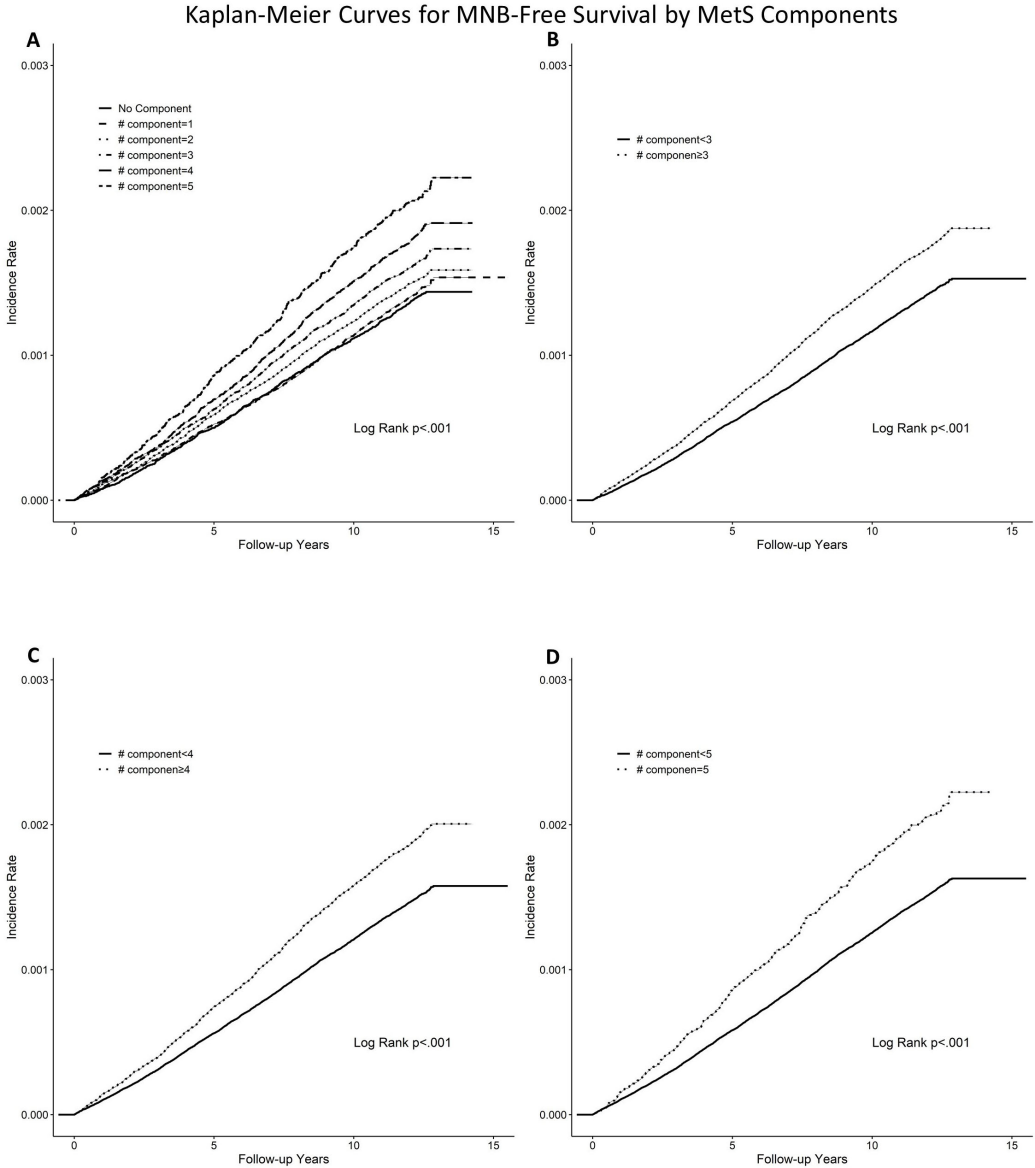
Cumulative malignant brain neoplasm-free survival by metabolic syndrome component burden. Kaplan-Meier curves illustrate MNB-free survival probability across the study follow-up period. **(A)** shows survival stratified by the number of individual metabolic syndrome (MetS) components (0–5). **(B–D)** present binary stratifications at thresholds of ≥3 versus <3, ≥4 versus <4, and ≥5 versus <5 components, respectively, corresponding to the adjusted hazard ratios reported in **Table 3**. Log-rank p<0.001 for all comparisons. MNB, malignant neoplasm of the brain; MetS, metabolic syndrome.

Kaplan-Meier survival analysis further illustrated the graded relationship between MetS burden and MNB-free survival over the study period (**Figure 1**). As shown in Panel A, cumulative survival curves diverged progressively with increasing MetS components, and the 5-component group had the lowest MNB-free survival probability throughout follow-up. All pairwise comparisons reached statistical significance by the log-rank test, consistent with the dose-response pattern observed in the Cox regression analyses. The binary stratification analyses (Panels B–D) revealed consistent separation in survival across all component thresholds. In the ≥3 versus <3 comparison (Panel B), Kaplan-Meier curves began to separate early in follow-up and maintained a progressively widening divergence through 12 years of observation, corresponding to a multivariate HR of 1.091 (95% CI: 1.017–1.170; p = 0.015). A comparable pattern of separation was observed in the ≥4 versus <4 comparison (Panel C), with the high-burden group exhibiting consistently lower MNB-free survival (HR: 1.131; 95% CI: 1.045–1.225; p = 0.002). The most pronounced visual separation was observed in the ≥5 versus <5 comparison (Panel D), corresponding to the highest adjusted hazard ratio among the threshold analyses (HR: 1.153; 95% CI: 1.040–1.278; p = 0.007). Collectively, these survival analyses provide visual corroboration of the stepwise increase in MNB risk associated with the accumulation of MetS components and reinforce the clinical relevance of cumulative metabolic burden as a determinant of brain tumor risk.

Assessing each MetS component as a mandatory inclusion criterion revealed differential effects on MNB risk. Among all components, elevated triglyceride levels were most strongly associated with MNB risk, with a multivariate HR of 1.517 (95% CI: 1.179–1.953; p = 0.001). This suggests that individuals with high triglyceride levels, regardless of other metabolic abnormalities, may have a substantially increased risk. Fasting blood glucose showed a weaker, non-significant adjusted HR of 1.050 (p = 0.537), despite being marginally significant in the univariate analysis, indicating its limited standalone contribution. The presence of a high waist circumference or hypertension showed a nuanced pattern. Waist circumference yielded an adjusted HR of 1.195 (p = 0.053), indicating a borderline association with risk, whereas hypertension, despite a significant univariate association, was not significant after adjustment (HR = 1.016; p = 0.781). HDL cholesterol had the weakest association among all variables, with a multivariate HR of 1.015 (p = 0.888), indicating negligible independent risk when accounting for other variables (**Table 4**). Taken together, these findings highlight triglyceride levels as the most robust individual metabolic risk factor for MNB, suggesting that not all components of MetS confer equal oncogenic potential.

## Discussion

In this large-scale nationwide cohort study, we investigated the association between MetS components and the risk of MNBs using comprehensive Korean NHIS data. The study findings demonstrate that the cumulative number of MetS components is positively associated with the risk of MNBs, although the magnitude of this association is modest. As presented in **Table 3**, participants with three or more MetS components had a statistically significant but modestly increased risk of brain malignancy (HR 1.091; 95% CI: 1.017–1.170), with hazard ratios incrementally increasing to 1.153 (95% CI: 1.040–1.278) for individuals with all five components. While these findings support the hypothesis that metabolic dysregulation may contribute to neuro-oncological risk, the relatively small effect sizes (HRs ranging from 1.091 to 1.153) suggest that MetS is likely one of multiple contributing factors rather than a dominant driver of brain tumor development.

Kaplan-Meier survival analysis provided complementary visual evidence reinforcing these findings (**Figure 1**). The progressive separation of MNB-free survival curves across all six component groups, and the consistent survival disadvantage demonstrated across the binary thresholds of ≥3, ≥4, and ≥5 components versus their respective counterparts (Panels B–D), closely mirror the dose-response pattern identified in the Cox regression models. Of particular note is the temporal character of curve divergence: in all three binary comparisons, survival separation emerged early in the observational period and was sustained throughout a mean follow-up exceeding 11 years, with no evidence of curve convergence at later time points. This temporally persistent pattern is consistent with the hypothesis that chronic, sustained metabolic dysregulation—rather than transient or reversible metabolic derangement—may exert a cumulative oncogenic influence over time. Although such findings cannot establish causality in an observational framework, the durability of the survival difference across more than a decade of follow-up provides biological plausibility for the notion that long-term metabolic burden contributes independently to the oncogenic milieu relevant to MNB development, and lends additional weight to the Cox regression findings reported above.

Previous studies have suggested that MetS promotes systemic inflammation, oxidative stress, altered metabolic signaling, and changes in immune responses, which could theoretically facilitate tumor development (6, 15). MetS creates a potentially high-risk environment through several interconnected mechanisms. Specifically, MetS induces chronic, low-grade inflammation, in which elevated pro-inflammatory cytokines may cause DNA damage and contribute to a tumor-permissive microenvironment (16–18). This is intensified by oxidative stress—a direct result of metabolic dysregulation—which may further damage DNA and promote cancer progression (8). Meanwhile, malignant cells undergo significant metabolic reprogramming, altering glucose and lipid metabolism to support rapid growth and survival (14, 19). Additionally, MetS may modulate immune function by affecting T cell metabolism, which plays a role in tumor surveillance (15). However, most mechanistic studies have been conducted *in vitro* or in specific tumor models, and the extent to which these mechanisms apply to the heterogeneous group of brain malignancies captured by ICD-10 codes C71.0–C71.9 remains uncertain.

Of particular clinical interest is the observation that elevated triglyceride levels appear to be the strongest individual MetS component associated with brain malignancy risk, even after multivariable adjustment (**Table 4**, HR 1.517; 95% CI: 1.179–1.953). This finding aligns with recent epidemiological studies linking serum triglycerides to high-grade gliomas, though the specificity of this relationship to particular histological subtypes remains incompletely characterized (20). Studies have shown that high triglyceride levels are associated with increased risk of certain brain tumors, independent of other metabolic factors. Additionally, research has demonstrated that lipid metabolism is altered in glioma biology, with some tumors exhibiting aberrant lipid storage and utilization (21, 22). Potential mechanisms linking hypertriglyceridemia and brain tumor development may include enhanced lipogenesis supporting membrane synthesis, altered inflammatory signaling, and metabolic reprogramming within tumor microenvironments (21–23). For example, studies have shown that triglycerides and their storage proteins are upregulated in some brain tumors, and their expression levels may correlate with tumor grade and patient prognosis (21, 24). However, whether elevated serum triglycerides directly promote gliomagenesis or serve as a biomarker of other metabolic alterations that drive tumorigenesis remains to be elucidated. Future mechanistic studies examining lipid transport across the blood-brain barrier, tumor cell lipid dependency, and the metabolic vulnerabilities of different brain tumor subtypes are needed to clarify these relationships (22, 25, 26).

These results suggest that managing metabolic health—particularly monitoring and controlling triglyceride levels—may inform MNB risk assessment in high-risk populations. However, given the modest effect sizes observed and the limitations discussed below, intensive lipid-lowering interventions specifically for brain tumor prevention should be approached cautiously until further validation studies are conducted. Nevertheless, these findings extend the clinical relevance of metabolic profiling beyond traditional cardiovascular and endocrine outcomes and may justify including metabolic parameters in population-based risk stratification frameworks.

Our study has several important limitations that warrant careful interpretation of these findings. First, malignant brain neoplasms in this study were identified using administrative ICD-10 codes (C71.0–C71.9) rather than pathologically confirmed diagnoses with detailed histological classification. Consequently, our outcome variable encompasses a heterogeneous group of primary brain malignancies, including glioblastoma, anaplastic astrocytomas, oligodendrogliomas, ependymomas, and other rare subtypes, each with potentially distinct etiologies, molecular profiles, and risk factor associations. We cannot determine whether the observed associations with MetS components are driven primarily by a single histological subtype (e.g., glioblastoma) or reflect a broader pattern across multiple tumor types. This fundamental limitation precludes definitive conclusions about histology- or molecular subtype-specific relationships between metabolic factors and brain malignancy risk. Future studies that integrate administrative data with cancer registry information containing detailed pathological and molecular tumor characterization are essential to address this critical knowledge gap.

Second, the potential for reverse causation is a significant concern in our study design. Although we implemented a one-year washout period by excluding participants diagnosed with MNB or who died within the first year of follow-up, this may be insufficient to fully eliminate reverse causation bias. Brain tumors often have a prolonged preclinical phase during which tumor-related metabolic alterations, changes in physical activity, psychological stress, or paraneoplastic phenomena may influence metabolic parameters. Elevated triglycerides, in particular, could reflect early tumor-related metabolic changes occurring months to years before clinical diagnosis rather than a true causal risk factor (27–29). Sensitivity analyses employing 2-year and 3-year exclusion windows, which represent methodological best practice for addressing this concern (30), were not conducted in the current study due to an institutional constraint inherent to Korean NHIS data governance: access to the database is granted on a strictly time-limited basis administered by HIRA, and our approved access period has concluded, rendering additional data queries legally and technically impossible. In lieu of formal sensitivity analyses, we note three indirect mitigating considerations: (1) the extremely low absolute MNB prevalence (0.14–0.20%) renders it statistically implausible that subclinical tumors meaningfully distorted population-level triglyceride distributions across a cohort of nearly 4 million individuals; (2) the temporally sustained divergence of Kaplan-Meier survival curves over more than a decade without convergence is inconsistent with a predominantly reverse causation-driven artifact; and (3) the graded dose-response pattern across metabolic burden strata is more consistent with a chronic systemic metabolic influence than with a temporally proximate reverse causation effect. Nevertheless, these arguments cannot substitute for formal lag period sensitivity analyses, and the possibility that elevated triglycerides partially reflect early tumor-related metabolic changes cannot be excluded. Future studies utilizing prospectively planned NHIS data access should pre-register sensitivity analyses with 2-year and 3-year exclusion periods as a priority analytical component.

Third, our analysis did not account for medication use, an important unmeasured confounder. Participants taking lipid-lowering medications (particularly statins), antihypertensive agents, or antidiabetic drugs would have different metabolic profiles than untreated individuals with similar underlying metabolic dysfunction. Regarding the directionality of bias: statin use would be expected to lower measured serum triglyceride levels in treated individuals, potentially underestimating the true association between underlying dyslipidemia and MNB risk (attenuation bias toward the null). Conversely, statins may exert pleiotropic anti-inflammatory and anti-proliferative effects that independently modulate cancer risk, the direction of which varies by tumor type and remains controversial (31, 32). Fibrate use would similarly reduce measured triglycerides in treated individuals. Metformin use, correlated with diabetes, may independently reduce cancer risk through AMPK-mediated pathways (33). Antihypertensive agents would analogously alter blood pressure measurements independently of underlying cardiovascular risk. The net direction of medication-related confounding is therefore complex and multidirectional. Importantly, the Korean NHIS database does contain prescription dispensing records that would enable adjustment for these medications; future studies with active NHIS data access should incorporate medication data as a priority covariate. We explicitly acknowledge that the triglyceride finding, while statistically robust, cannot be interpreted as causal evidence without medication adjustment. Incorporating medication data in future analyses would substantially improve the validity of findings.

Fourth, the modest magnitude of the observed effect sizes (HRs 1.091–1.517) raises questions about clinical significance despite statistical significance. While population-level associations of this magnitude may have public health implications given the high prevalence of MetS, the individual-level risk increase is relatively small. For comparison, established strong risk factors for other cancers typically show hazard ratios of 2.0 to 3.0. The clinical utility of metabolic risk stratification for brain tumor prevention requires validation through cost-effectiveness analyses and by assessing whether metabolic interventions can meaningfully reduce incidence rates in high-risk populations. Furthermore, unmeasured confounding by factors not captured in our dataset (e.g., dietary patterns, specific occupational exposures, genetic susceptibility variants) could account for a substantial portion of the observed associations.

Fifth, our cohort consisted exclusively of Korean adults aged 40 years or older who participated in national health screening programs. The prevalence and clustering of MetS components, as well as brain tumor epidemiology, vary substantially across ethnic groups, genetic backgrounds, environmental exposures, and healthcare systems. Korean individuals tend to develop metabolic complications at lower body mass indices than Western populations (34), and genetic variants that influence lipid metabolism and cancer susceptibility differ across ancestries. Additionally, dietary patterns (e.g., high sodium intake, fermented foods), occupational exposures, and environmental risk factors unique to Korea may modify the MetS-brain tumor relationship in ways that do not generalize to other populations. Therefore, direct extrapolation of our findings to non-Korean, younger, or ethnically diverse populations requires considerable caution. Multiethnic validation studies across diverse geographic regions are essential to determine the generalizability of these associations and to identify potential population-specific risk thresholds.

Sixth, although our study benefits from comprehensive nationwide coverage through the NHIS system, residual confounding by socioeconomic status, healthcare access patterns, and health-seeking behaviors cannot be entirely ruled out. Individuals participating in regular health screenings may systematically differ from non-participants in ways that relate to both metabolic health and cancer detection rates. Additionally, variations in diagnostic practices, neuroimaging availability, and referral patterns across regions and healthcare facilities could introduce heterogeneity in case ascertainment. Finally, our analysis did not incorporate information on tumor molecular markers, genetic mutations (e.g., IDH1/2, MGMT methylation, 1p/19q codeletion), or other molecular classifiers that have become central to brain tumor classification (35). These molecular features strongly influence prognosis and may be associated with distinct etiological pathways. Whether metabolic risk factors differentially affect molecularly defined tumor subgroups remains unknown and represents a critical area for future investigation.

Despite these limitations, our findings have several important implications for clinical practice and future research. In aging populations with rising burdens of metabolic disease, routine screening and management of metabolic risk factors may confer benefits beyond traditional cardiovascular and diabetes outcomes. Our results suggest that lipid profile monitoring, particularly of triglyceride levels, could contribute to brain tumor risk assessment frameworks, though individualized evaluation that accounts for population-specific patterns is essential. However, any clinical recommendations for metabolic interventions specifically targeting brain cancer prevention would be premature without prospective validation studies demonstrating that metabolic optimization actually reduces incidence rates.

Future research should prioritize several key objectives. First, linking administrative databases to detailed cancer registries that include pathological, molecular, and treatment data would enable histology- and molecular subtype-specific analyses critical for understanding etiological heterogeneity. Second, prospective cohort studies with serial metabolic assessments and longer lag periods can better address concerns about reverse causation and establish temporal relationships. Third, investigating medication effects and potential gene-environment interactions would refine risk estimates. Fourth, multiethnic validation studies across diverse populations are essential for assessing generalizability and identifying population-specific risk modifiers. Fifth, mechanistic studies examining how systemic metabolic alterations influence brain tumor initiation and progression at the molecular level would provide biological plausibility and identify potential intervention targets.

In conclusion, this large-scale nationwide cohort study demonstrates that cumulative metabolic syndrome burden is associated with a modest but statistically significant increase in the risk of malignant brain neoplasms, with elevated triglycerides showing the strongest individual component association. While these findings suggest that metabolic health may have implications for neuro-oncological risk assessment, the modest effect sizes, potential for reverse causation, inability to distinguish histological subtypes, and population-specific nature of our cohort necessitate cautious interpretation. These results provide a foundation for future mechanistic investigations and prospective validation studies across diverse populations aimed at elucidating the complex relationships between metabolic dysregulation and the development of brain malignancies. It must be emphasized that the observed associations cannot be attributed to any specific histological or molecular subtype, as the outcome variable aggregates diverse tumors under ICD-10 codes C71.0–C71.9. The triglyceride association may reflect the biology of a single dominant subtype such as glioblastoma, or a broad metabolic susceptibility shared across subtypes; these possibilities cannot be distinguished with the current data. No mechanistic inference is warranted from the present findings without subtype-stratified analyses using integrated administrative-registry databases.

## Data Availability

The original contributions presented in the study are included in the article/supplementary material. Further inquiries can be directed to the corresponding authors.
